# Development and validity testing of a matrix to evaluate maturity of clinical pathways: a case study in Saskatchewan, Canada

**DOI:** 10.1186/s12913-024-11239-x

**Published:** 2024-07-10

**Authors:** Crystal Lynn Larson, Jason Robert Vanstone, Taysa-Rhea Mise, Susan Mary Tupper, Gary Groot, Amir Reza Azizian

**Affiliations:** 1 Clinical Excellence, Saskatchewan Health Authority, Regina, SK Canada; 2 College of Medicine, University of Saskatchewan, Saskatoon, SK Canada

**Keywords:** Clinical pathways, Maturity matrix, Quality improvement, Evaluation, Measurement

## Abstract

**Background:**

Healthcare systems are transforming into learning health systems that use data-driven and research-informed approaches to achieve continuous improvement. One of these approaches is the use of clinical pathways, which are tools to standardize care for a specific population and improve healthcare quality. Evaluating the maturity of clinical pathways is necessary to inform pathway development teams and health system decision makers about required pathway revisions or implementation supports. In an effort to improve the development, implementation, and sustainability of provincial clinical pathways, we developed a clinical pathways maturity evaluation matrix. To explore the initial content and face validity of the matrix, we used it to evaluate a case pathway within a provincial health authority in Saskatchewan, Canada.

**Methods:**

By using iterative consensus-based processes, we gathered feedback from stakeholders including patient and family partners, policy makers, clinicians, and quality improvement specialists, to rank, retain, or remove enablers and sub-enablers of the draft matrix. We tested the matrix on the Chronic Pain Pathway (CPP) for primary care in a local pilot area and revised the matrix based on feedback from the CPP development team leader.

**Results:**

The final matrix contains five enablers (i.e., Design, Ownership and Performer, Infrastructure, Performance Management, and Culture), 20 sub-enablers, and three trajectory definitions for each sub-enabler. Supplemental documents were created for six sub-enablers. The CPP scored 15 out of 40 possible points of maturity. Although the pathway scored highest in the Design enabler (10/12), it requires more attention in several areas, specifically the Ownership and Performer and the Performance Management enablers, each of which scored zero. Additionally, the Infrastructure and Culture enablers scored 2/4 and 3/8 points, respectively. These areas of the CPP are in need of improvement in order to enhance the overall maturity of the CPP.

**Conclusions:**

We developed a clinical pathways maturity matrix to evaluate the various dimensions of clinical pathways’ development and implementation. The goals of this initial work were to develop and validate a tool to assess the maturity and readiness of new or existing pathways and to track pathways' revisions and improvements.

**Supplementary Information:**

The online version contains supplementary material available at 10.1186/s12913-024-11239-x.

## Background

Healthcare systems are transforming to become learning health systems (LHS) in which quality care and value are achieved by demonstrating improvements to patient experiences and health outcomes, provider experiences, population health, and health system costs [1]. In LHS, continuous improvement is achieved by data-driven and research-informed approaches [1–5]. One of these approaches is the utilization of clinical pathways [6, 7]. Clinical pathways, also known as critical or integrated care pathways [8], are operationally defined as tools to standardize care for a specific population, translate guidelines or evidence into local structure, create a structured multidisciplinary care plan, and detail a care plan in an inventory of actions [9–12]. Clinical pathways can improve patients' and providers' experience and satisfaction, resource utilization, and inter-professional teamwork while reducing knowledge transition gaps, healthcare team burnout, costs, and variation in care [11, 13–15]. Further, clinical pathways can be utilized to improve the domains of healthcare quality including safety, effectiveness, patient-centeredness, timeliness, efficiency, and equitability [14, 16, 17].

Implementation barriers such as knowledge users’ awareness, stakeholders’ engagement, information technology (IT) infrastructure, and performance management have been shown to impede optimal integration of clinical pathways into healthcare systems [11–13, 17–19, 20]. To increase their impact, the development and implementation of clinical pathways should be guided by theories, models, or frameworks [12, 21]. Despite outlining the development and implementation of clinical pathways, many frameworks do not specify how to evaluate the maturity of pathways. For our purposes, we defined maturity as a dynamic state of planning, development, and readiness for a pathway to be implemented, replicated or scaled up, and sustained in its intended clinical settings in which the goals or outcomes of the pathway are achieved. Evaluating the maturity of clinical pathways can inform pathway development teams and health system decision makers about required pathway revisions or implementation supports to improve implementation outcomes such as acceptability, fidelity, feasibility, adoption, appropriateness, and sustainability [22]. Further, evaluating the maturity of clinical pathways enhances the effectiveness of clinical pathways by ensuring they are functioning as intended and achieving the planned effects at the patient, provider, and system levels [11]. To our knowledge, only one paper has been published that describes a formal and standardized process to evaluate the maturity of clinical pathways [13]. Although the maturity model described by Schriek et al. [13] provides a foundation for pathway evaluation, the Saskatchewan Health Authority (SHA) Clinical Pathways Core Team (CPCT) aimed to ensure that the purpose of the model, its enablers and sub-enablers and their definitions, and their trajectory definitions are compatible with the SHA environment. The process and results of verifying content and face validity of the proposed matrix through key stakeholders’ engagement and testing the matrix with a case pathway prototype within the SHA in Canada are described.

## Methods

### Setting

Serving a diverse population of 1.2 million residents with over 45,000 employees and physicians, the SHA is responsible for delivery of the majority of publicly funded health services throughout the province of Saskatchewan [23]. The SHA was launched in December 2017 through the amalgamation of 12 former health regions. The Clinical Excellence portfolio of the SHA is responsible for the development, implementation, and evaluation of new clinical pathways that guide clinical care for targeted conditions [24]. The SHA currently has clinical pathways for Acute Stroke, Bariatric Surgery, Chronic Pain, Fertility Care, Hip and Knee Replacement Surgery, Lower Extremity Wounds, Multiple Sclerosis, Pelvic Floor, Prostate Cancer, and Spine [24]. Additionally, there are pathways for Diabetes, Chronic Obstructive Pulmonary Disease (COPD), and Long COVID that are in development (Table 1). These pathways have been or are being developed by multidisciplinary stakeholder teams consisting of operational leaders, clinical experts, and patient and family partners (PFPs) [24].

**Table 1:** List of clinical pathways in Saskatchewan

Name	Development Date	Developed by SHA (including former health regions) or MoH^a^	Status
Bariatric Surgery	2009	MoH	Fully Developed But Implemented in One Local Setting^b^
Hip and Knee Replacement Surgery	2009	MoH	Fully Developed and Implemented Provincially^b^
Spine	2010	MoH	Fully Developed and Implemented Provincially
Pelvic Floor Care	2012	MoH	Fully Developed and Implemented Provincially
Prostate Cancer	2012	MoH	Fully Developed and Implemented Provincially
Fertility Care	2015	MoH	Fully Developed and Implemented Provincially
Lower Extremity Wounds	2016	MoH	Fully Developed and Implemented Provincially
Acute Stroke	2017	MoH	Fully Developed and Implemented Provincially
Multiple Sclerosis	2019	MoH	Fully Developed and Implemented Provincially
Chronic Pain	2022	SHA	Fully Developed but Implemented in One Local Setting
Chronic Obstructive Pulmonary Disease (COPD)	TBD^c^	SHA	Under Development^b^
Diabetes	TBD	SHA	Under Development
Long COVID	TBD	SHA	Under Development

^a^SHA, Saskatchewan Health Authority; MoH, Saskatchewan Ministry of Health

^b^Fully Developed and Implemented Provincially indicates the pathway is no longer in the development phase and has been implemented across Saskatchewan. The pathway is monitored and modified as new evidence and best practice emerge; Fully Developed but Implemented in One Local Setting indicates that the pathway is no longer in the development phase but has not been implemented across Saskatchewan; Under Development indicates the pathway is in the development phase and has not been implemented

^c^TBD, To Be Determined

In 2021, nine pathways developed by the Saskatchewan Ministry of Health (MoH) were transitioned to the SHA, for a total of 13 pathways that fall within SHA accountability (Table 1). At a provincial level, this accountability includes the responsibility of supporting development and implementation, maturing of clinical pathways, and progress reporting to the MoH. As the former MoH pathways were developed without a standardized approach, they varied in their design and scale (provincial versus local settings). Gaps were recognized in that no processes, tools, or methods existed to validate the maturity of each pathway, to compare the pathways to one another, and to provide progress reporting to the MoH. The SHA CPCT planned to develop a maturity evaluation matrix to bridge these gaps by providing a tool that could measure levels of maturity via design, awareness, usage, metrics inclusion, owner engagement and participation, and provincial replicability of the clinical pathways.

### Developing the maturity matrix

A search of published English language literature in MEDLINE via Pubmed, CINAHL, Cochrane Library, and Google Scholar for maturity evaluation matrices or models for clinical pathways resulted in identification of only one relevant publication [13]. The maturity matrix published by Schriek et al. [13] contained five enablers and 19 weighted sub-enablers with four trajectory definitions (low, moderate, high, and top) for each sub-enabler. The matrix was initially examined and evaluated by the SHA CPCT to determine its compatibility within the specific context of Saskatchewan. Our CPCT included members with various backgrounds (medicine, quality improvement, implementation science, learning health systems, research, and psychology) as well as a pathway development team leader. The assessment of Schriek et al.’s matrix revealed the need to modify it based on the current knowledge in the fields of quality improvement, implementation science, and evaluation. For example, sub-enabler definitions and their trajectories required revisions to improve their clarity, potential translation issues were addressed and language was refocused to be patient-centered, a dedicated sub-enabler was added to capture the intricacies of the patient journey, and the complexity of the maturity scoring was simplified allowing evaluators to better distinguish between levels.

**Fig. 1:**
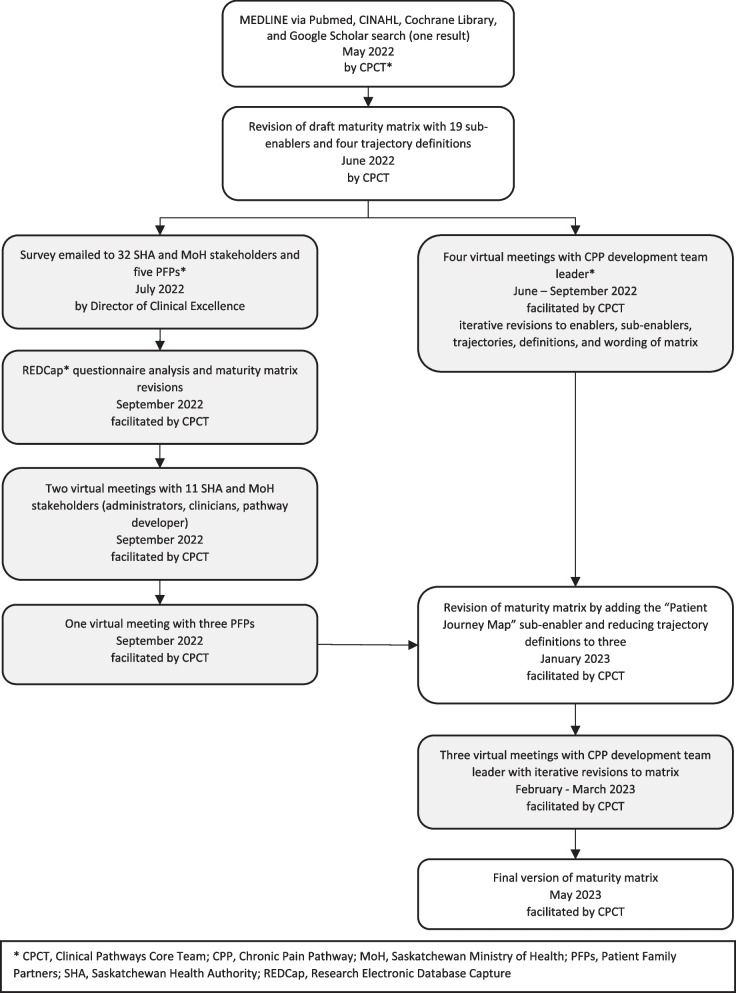
Development of maturity matrix and iterative consensus-based processes

Using an iterative consensus-based process, email invitations (one initial and one reminder email two weeks later) were sent to SHA and MoH stakeholders with differing levels of experience in clinical pathway development and implementation as well as PFPs. Both emails were sent from the Director of Clinical Excellence in July 2022 with an attached copy of the draft maturity matrix (Fig. 1). We used purposeful and snowball sampling methods to identify the stakeholders from SHA and MoH. They came from diverse disciplines within the SHA, including nursing, executive directors, managers, clinical department heads, physicians, administrators, and a pathway developer from the MoH. Knowledge of pathway development among stakeholders ranged from those that had been involved with development and utilization of pathways to those that had moderate to no exposure in this area. To identify the PFPs, we asked the SHA or MoH stakeholders to recommend PFPs who they had previously worked with as well as contacted the SHA’s Patient and Client Experience (PCE) department [25, 26]. All PFPs were registered with the SHA’s PCE department and were compensated as per the organization’s PFP policy [26, 27]. Knowledge of pathway development among the PFPs ranged from involvement with pathway development and related concepts to no previous exposure in this area. Beyond diversity in professional roles, the stakeholders and PFPs included individuals of different ages, genders, ethnicities, and immigration backgrounds, reflecting a range of lived experiences and perspectives.

The stakeholders and PFPs were asked to review the draft maturity matrix, determine which enablers and sub-enablers of the matrix should be kept and weighted more importantly on a 10-point Likert scale, choose which sub-enablers to be removed, and complete a REDCap (Research Electronic Database Capture) questionnaire (Supplementary file 1) [28, 29]. Sub-enablers with a mean of 7 to 10 were considered important for inclusion.

After receiving the feedback, all potential participants were invited to attend virtual follow-up meetings in September 2022 via Webex platform [30]. Participation was voluntary and no identifiable information was collected during the virtual meetings (Fig. 1).

During the sessions with the SHA and MoH stakeholders, questions were posed to participants about the inclusion of categories integral to pathway development, including pathway ownership (i.e., owner identity), patient involvement (e.g., ongoing stakeholder engagement), provincial integration (e.g., network of pathways) and replication (e.g., capacity monitoring). Content and face validity related to the relevance, appropriateness, and utility of the tool were explored and verified through discussions regarding the purpose of the tool and potential end users.

The session with PFPs had a series of seven questions seeking patients’ perspectives (five general questions and two questions related to the importance of sub-enablers) (Table 2). The questions were designed in consultation with the SHA’s PCE department and based on the SHA’s “Setting the stage for successful meetings with patient family partners (PFPs)” guidelines (internal document). Highlights of the guidelines include building in sharing time (ice breakers, stories), avoiding medical jargon or acronyms, and listening to PFPs stories, even if they are about care that did not go well. We started with open ended questions about PFPs’ experience in pathways or Saskatchewan’s health system and asked questions related to what is important to them in their care (i.e., pathway outcomes). To ensure the use of plain language and avoid using jargon, the CPCT conducted a readability analysis on the matrix. Results indicated that the matrix was at the university graduate level on the Flesch Readability Scale. Given this, the CPCT decided to focus on overarching concepts instead of one by one sub-enabler review. This was done to promote PFP’s engagement in an open and inviting discussion.

**Table 2:** List of questions asked during a session with patient and family partners

Type of Question	Question
General	Have you ever heard of the term “Clinical Pathways” or been involved in Clinical Pathway work?
General	From a patient perspective, what are the most important outcomes (measures, metrics) of the pathways? What outcomes would indicate successful pathways?
General	From a patient perspective, what are some indicators of a ‘good’ pathway? What are the things that make you confident you are receiving appropriate care? (appropriate could mean streamlined or seamless, clear communication, patients’ preferences considered in treatment, shared decision making, patients’ concerns addressed)
General	Are there any barriers that you have experienced when engaging in Clinical Pathway development or SHA activities? Any facilitators?
General	What are some challenges you have noticed in the healthcare system? (So that we can factor these into pathways)
Sub-enabler related	When looking at the list of enablers and sub-enablers, are there any that seem most important? Or, are there any that don’t seem important at all?
Sub-enabler related	Stakeholder engagement, owner identity, and metrics. For these three sub-enablers we would ask for more feedback. What are your thoughts on this? What would success in these areas look like?

Notes were taken during the meetings and summarized to participants who then provided additional feedback or context and validated the summary.

It is important to note that, while we chose to begin testing the use of the maturity matrix after the above rounds of development and refinement, the document is intended to be dynamic and continuously evolving based on feedback, context, and experience. While we have finalized the tool for current use, we remain open to future revisions to ensure its efficacy in improving pathways. The current goal is to maintain stability for a period of time to facilitate practical application, testing, and evaluation.

### Applying the maturity matrix

We chose the Chronic Pain Pathway (CPP) because it was a newly developed pathway that had not been put into practice across the province. Using the Webex platform, the CPCT and the CPP development team leader met virtually from June to September 2022 (first round) and used the draft matrix to evaluate the CPP. The draft matrix contained 19 sub-enablers, each with four trajectories (low, moderate, high, and top). The purpose of the first round evaluation was to focus on the utility, clarity, and applicability of the draft matrix’s various components and scoring definitions (Fig. 1). From February to March 2023 (second round), the CPCT asked the pathway leader for her input on the elements of the revised maturity matrix, which contained 20 sub-enablers with three trajectories (low, moderate, and high). For this round of evaluation, we wanted to know if the terminology was relevant and if the matrix could be effectively used to rate the development of the pathway.

Based on our experience, we recommend the assembly of a diverse assessment team comprising of individuals with various backgrounds to complete a clinical pathway evaluation. This team may include patient and family partners, clinical experts, pathway developers, administrators, operational staff, quality improvement experts, implementation experts, researchers, administrative personnel, information technology experts, and policy makers. Assessments should adopt an iterative process of current state assessment and should be conducted regularly (e.g., annually) through a series of collaborative meetings where team members systematically review components of the pathway matrix and discuss the status of the pathway for each sub-enabler based on data collected over the year. Supplementary documents should be reviewed for relevance and teams should score the sub-enablers based on the defined trajectories. In the event of a disagreement among team members regarding a particular score, we recommend employing consensus-based approaches to resolution, ensuring that divergent viewpoints are acknowledged and reconciled through constructive dialogue.

## Results

### Design and structure of the maturity matrix

Thirty-seven people (32 SHA and MoH stakeholders and five PFPs) were invited to participate via email. Fourteen responses were received from the REDCap questionnaire (response rate = 38%). The mean score for importance of all sub-enablers was 7.9/10 (mean range: 7 – 9, SD: 1.8).

Two virtual follow-up meetings (two hour sessions) with 11 SHA and MoH stakeholders (all 32 stakeholders were invited) and one (two hour session) with three PFPs (all five PFPs were invited) were held. During the follow-up meetings with SHA and MoH stakeholders, the attendees emphasized the importance of and equal weighting for all sub-enablers in the maturity matrix, resulting in the inclusion of all in the final matrix. During the follow-up meeting with PFPs, there was agreement that all sub-enablers were of equal relevance. The importance of categories representing patient preferences was highlighted, including multidisciplinary care (Ongoing Stakeholder Engagement sub-enabler), standardization in care (Design Approach sub-enabler), evidence based approaches (Compliance sub-enabler), and ease of navigation (Clarity in the Decision Criteria sub-enabler), all of which had been considered during the development of the matrix.

Based on the feedback received during the follow-up sessions, the CPCT added a “Patient Journey Map” sub-enabler under the “Design” enabler (resulting in 20 sub-enablers), reduced trajectory categories to three (low, moderate, and high), rearranged the order of sub-enablers, and modified the definitions of two enablers and eight sub-enablers to align with the needs of the SHA context (Table 3). A full list of maturity matrix enablers, sub-enablers, their definitions, and the three trajectory definitions are presented in Supplementary file 2.

**Table 3:** Summary of maturity matrix enablers, sub-enablers, and their definitions

Enabler	Sub-Enabler	Definition	Modified From Original^a^
**Design**	Pathway Objective Alignment	The degree by which the objective of the pathway is aligned to the objective of the care delivery to the specific patient group of the pathway	No
	Pathway Definition	The degree in which the design of the pathway is defined with a clear structure, terminology, and roles	No
	Compliance	The degree in which a pathway is designed, taking into consideration integrated policies, best clinical practice guidelines, evidence, and legislation	Yes
	Clarity in the Decision Criteria	There is sufficient detail in the decision moments and in the decision criteria in the design of the pathway	Yes
	Patient Journey Map^b^	There is sufficient detail that has been included in the patient journey map or algorithm that outlines all of the patient touchpoints that occur within the pathway	Not Applicable
	Design Approach	The degree in which a structured approach (e.g., a reference framework) is used and different stakeholders were involved during the design of the pathway (from primary care to specialized / hospital based care)	Yes
**Owner and Performer**	Owner (Identity)	The extent to which the pathway ownership structure is effective in improving the pathway performance	No
	Role Awareness/Role Functionality	The degree in which a pathway participant has awareness of his/her part in the pathway and the ability to perform his/her task as described in the pathway design	Yes
**Infrastructure**	IT Infrastructure and Information Sharing	The degree by which IT infrastructure facilitates the sharing of materials and information across both internal and external data systems	Yes
	Network of Pathways	The degree to which a pathway is interconnected to other pathways that have overlapping clinical problems	Yes
**Performance Management**	Metrics Alignment	The degree in which pathway metrics (i.e., process, outcome, and balancing measures) are uniformly defined, and pathway objectives (e.g., patient and provider experience) have been considered in the development of the metrics	Yes
	Structured Collection of Data	The degree in which a structured data collection plan is in place (including what is measured, in which setting, how will it be measured, by whom and by when [frequency and timeframe])	Yes
	Availability/Accessibility of Data	The degree in which the availability and accessibility of pathway data facilitates the development of metrics	Yes
	Metrics Use	The degree in which the pathway metrics (i.e., process, outcome, and balancing measures) are effectively used to improve the achieved performance	Yes
	Availability of Performance Information	The degree in which pathway metrics (i.e., process, outcome, and balancing measures) are available, shared, and translated into something that stakeholders can understand	Yes
	Capacity Monitoring	The degree by which there is adequate allocations of key resources, such as facilities, equipment, and human resources, and these allocations are monitored	Yes
**Culture**	Pathway Awareness	The degree in which mechanisms are in place to raise stakeholders' (e.g., patients, clinicians, etc.) awareness of the pathway	Yes
	Ongoing Stakeholder Engagement	The degree in which stakeholders, including patient partners, are engaged to provide ongoing contributions for change to improve the pathway structure and its processes	Yes
	Adaptability	The degree in which the pathway is designed with the adaptability for implementation and replication across multiple settings and changes are tracked within each setting when this occurs	Yes
	External Maturity Evaluation	The degree in which the pathway is audited for maturity by an independent SHA governing body	Yes

^a^Original definitions are adapted from Schriek et al. [13]

^b^Patient Journey Map was added as a new sub-enabler to the maturity matrix

Since all sub-enablers of the maturity matrix were considered to be of equal importance by the stakeholders and PFPs, we did not incorporate weighting the sub-enablers as had been done in the maturity matrix that we modelled our work on [13]. Therefore, we used a simple sum of the sub-enablers’ maturity levels (low = 0, moderate = 1, and high = 2) to score maturity of a pathway. This results in minimum and maximum scores of 0 and 40, respectively.

During the revisions of the maturity matrix by the CPCT and the feedback received from stakeholders, the need was identified to develop supplemental documents for six sub-enablers (Pathway Objective Alignment, Compliance, Design Approach, Network of Pathways, Capacity Monitoring, and Adaptability). These documents were either adapted from other sources (e.g., SHA’s measurement planning templates), or templates were developed ad hoc (Table 4). The supplemental document templates are provided in Supplementary files 3 to 8.

**Table 4:** Summary of maturity matrix enablers and sub-enablers with supplemental documents

Enabler	Sub-Enabler	Supplemental Document
**Design**	Pathway Objective Alignment	Clinical Pathway Alignment Tool
	Compliance	Clinical Pathway Development Record
	Design Approach	Clinical Pathway Prototype Checklist
**Infrastructure**	Network of Pathways	Clinical Pathway Listing
**Performance Management**	Capacity Monitoring	Capacity Monitoring Mural Board^a^
**Culture**	Adaptability	Replication Documentation Checklist

^a^Mural is a virtual board and workspace for teams to collaborate visually [31]. For the purpose of this manuscript, a Word document version of the Mural board is provided as a supplementary file

### Scores of chronic pain pathway evaluation

The CPP development team leader and the CPCT met virtually in two rounds of meetings (i.e., seven meetings total) (Fig. 1) and scored the pathway twice. The first round of CPP scoring (four meetings, 6.5 h in total) resulted in a score of 18/57 (19 sub-enablers with four trajectory definitions [low = 0, moderate = 1, high = 2, and top = 3]) (Table 5). The CPP scored highest in Design (11/15), followed by Culture (4/12), Infrastructure (1/6), Owner and Performer (1/6), and Performance Management (1/18). The score was 15/40 in the second round (three meetings, 4.5 h in total), with the highest score in Design (10/12), followed by Infrastructure (2/4), Culture (3/8), Owner and Performer (0/4), and Performance Management (0/12) (Table 5).

**Table 5:** Chronic pain pathway first and second rounds scores, and reasons for score differences

Enabler	Sub-enabler	CPP^a^ Scores – First Round^b^	CPP Scores – Second Round^c^	Reasons or Rationale for Score Difference
**Design**	Pathway Objective Alignment	3 (Top)	2 (High)	The scale was changed from 4 to 3 trajectory definitions, which resulted in a change in score from 3 to 2. This change in score reflects changes in the matrix
	Pathway Definition	1 (Moderate)	2 (High)	For the second round of scoring, clearer trajectory definitions were developed. In addition, the development team had worked on clarifying the scope and expectations of the pathway with greater precision. The change in score reflects both changes in the pathway and changes in the matrix scoring definitions
	Compliance	3 (Top)	0 (Low)	In the first round, receiving a score of 3 (top) required the team to subjectively rate whether the pathway took into account policies, best practice guidelines, and evidence. In the second round, efforts were made to address the subjectivity of this rating. The matrix development team created a Clinical Pathway Development Record in which pathway development teams were required to document the sources used for evidence verification. During the second round of scoring, the CPP pathway developers had not yet completed the new supplemental document, resulting in a score of zero (0) for non-completion. This change in score reflects a change in the objectivity of the requirements for achieving a top score
	Clarity in the Decision Criteria	1 (Moderate)	2 (High)	In the first round of scoring, a Patient Journey Map was required, but the CPP developers had not yet completed this, resulting in a score of 1. However, the Patient Journey Map was considered an essential element that should be treated as a distinct sub-enabler. The Patient Journey Map was completed prior to the second round of scoring, resulting in a higher score for this sub-enabler in the second round
	Patient Journey Map^d^	-	2 (High)	The Patient Journey Map was added as a sub-enabler between the first and second round and the CPP team created a Patient Journey Map, which resulted in a higher score
	Design Approach	3 (Top)	2 (High)	The scale was changed from 4 to 3 trajectory definitions, which resulted in a change in score from 3 to 2. This change in score reflects changes in the matrix
**Owner and Performer**	Owner (Identity)	1 (Moderate)	0 (Low)	In the first round, the CPP owners were identified. In the second round, the SHA’s organizational restructure resulted in vacancies for sponsor and owner roles, and the pathway scored lower due to the absence of ownership
	Role Awareness/Role Functionality	0 (Low)	0 (Low)	There was no variation in the scores
**Infrastructure**	IT Infrastructure and Information Sharing	0 (Low)	0 (Low)	There was no variation in the scores
	Network of Pathways	1 (Moderate)	2 (High)	In the first round, the Clinical Pathway Listing template was finalized, but the template lacked the desired level of rigor as it had not been fully operationalized. However, by the second round, the template had been fully developed and completed by the CPP team, meeting the desired standards, resulting in a higher score
**Performance Management**	Metrics Alignment	0 (Low)	0 (Low)	There was no variation in the scores
	Structured Collection of Data	1 (Moderate)	0 (Low)	In the first round, the CPP pathway scored 1, indicating that a data collection plan was in development. However, by the second round of scoring, the prioritization of the data collection plan had changed due to the SHA organizational restructuring. This resulted in a reduction in the score
	Availability/Accessibility of Data	0 (Low)	0 (Low)	There was no variation in the scores
	Metrics Use	0 (Low)	0 (Low)	There was no variation in the scores
	Availability of Performance Information	0 (Low)	0 (Low)	There was no variation in the scores
	Capacity Monitoring	0 (Low)	0 (Low)	There was no variation in the scores
**Culture**	Pathway Awareness	1 (Moderate)	1 (Moderate)	There was no variation in the scores
	Ongoing Stakeholder Engagement	2 (High)	2 (High)	There was no variation in the scores
	Adaptability	1 (Moderate)	0 (Low)	In the first round, trajectory definitions were based on subjective assessments. In the second round, a Replication Documentation Checklist was developed and introduced as an objective requirement. The CPP development team had not yet completed this, which resulted in a lower score
	External Maturity Evaluation	0 (Low)	0 (Low)	There was no variation in the scores
**Total Score**		18/57	15/40	

^a^*CPP* Chronic Pain Pathway

^b^The maturity matrix with 19 sub-enablers and four trajectory definitions (low = 0, moderate = 1, high = 2, and top = 3) assigned to each sub-enabler was utilized during the first round of CPP scoring

^c^The maturity matrix with 20 sub-enablers and three trajectory definitions (low = 0, moderate = 1, and high = 2) assigned to each sub-enabler was utilized during the second round of CPP scoring

^d^Patient Journey Map sub-enabler was added prior to the second round of CPP scoring

## Discussion

We developed a maturity evaluation matrix for clinical pathways based on a previously published matrix in which a generic business process maturity model was utilized [13]. We refined the previous matrix using iterative consensus-based processes that included a questionnaire and multiple group discussions with PFPs, policy makers, clinicians, and quality improvement specialists. All enablers from the previous matrix were retained, but 16/19 sub-enablers were modified and one sub-enabler (i.e., Patient Journey Map) was added.

The existing literature on this topic is limited, which has underscored a significant gap concerning the absence of a comprehensive tool for evaluating the maturity of clinical pathways. This proposed maturity matrix is specifically designed to support clinical pathway development and implementation teams in assessing various aspects of pathway maturity. These aspects include a) Pathway design: This includes factors such as clinical components, objectives of care delivery, adherence to evidence-based practices, and the extent of stakeholder involvement; b) Ownership: This category involves aspects such as leadership engagement and involvement, the assessment of role awareness, and the functionality of different roles within the pathway; c) Infrastructure: This pertains to the integration of infrastructure, both internally and externally, for disseminating information and the connectivity of the pathway to other relevant pathways; d) Performance Management: This encompasses the selection and utilization of metrics, the availability and collection of data, data usage, and planning for provincial replication; and e) Culture: This focuses on elements like pathway awareness, stakeholder engagement, the capability for provincial implementation, and the audit and evaluation process.

To facilitate this evaluation, completion of the supplemental documents contained within the matrix is required. For instance, the Clinical Pathway Listing document aids in assessing whether connections or overlap with other pathways were considered during the development and implementation of the pathway currently under review.

By using our proposed scoring tool, a clinical pathway development team can compare the score of the pathway with previous scores to ensure that the score is improving over time. Further, health system decision makers are able to compare different pathways or examine low scores for commonalities amongst multiple pathways to identify resource needs and systemic issues. For example, if all pathways score low in the Owner (Identity) sub-enabler, it may indicate sponsorship constraints for clinical pathways in an organizational structure that may impact the sustainability of pathways. Further research is needed to understand the interpretation of the total score and whether a threshold score can be identified for satisfactory maturity. At present, pathway development teams are encouraged to make decisions based on individual sub-enabler scores and to use total scores as an overall measure of pathway maturity.

It is important for all healthcare interventions to incorporate aspects of equity, diversity, and inclusion (EDI) into their development and implementation. While these are not explicitly sub-enablers in the current version of the maturity matrix, several components within the matrix incorporate considerations for EDI. For example, the patient journey map, provincial service planning (using supplemental tools such as capacity monitoring), and engagement of diverse patient and family partners and a multidisciplinary team for pathway development inherently encompass aspects of EDI. As future iterations of the maturity matrix are evaluated and modified, inclusion of EDI as a specific sub-enabler may be considered.

Our evaluation showed areas in which the CPP can be improved as well as areas that the matrix can guide further development of the CPP. For example, the CPP scored highest in Design, which may reflect the status of the pathway during the assessment. The CPP has been fully developed but only implemented within one local setting, with plans to be implemented provincially. In addition, the pathway was scored during a time of leadership change within the SHA, leaving a temporary gap in pathway ownership. This status impacted the pathway’s scores for elements such as sponsorship, owner identity, role awareness, connectivity, data collection, provincial replication, and ongoing adaptability. Low scores in the CPP infrastructure and performance management may reflect gaps in organizational investment in resources to support implementation and evaluation of clinical pathways.

During the follow-up sessions, the participants indicated that all sub-enablers held equal significance. This differs from the findings of Schriek et al. wherein weights were incorporated into the analysis through stakeholder consultations [13]. The difference in weighting may be attributed to various factors, such as the revisions we made to the original maturity matrix, differences in methodological approaches (our study's utilization of the consensus-based approach versus the Delphi approach employed by Schriek et al.), and variations in the stakeholders involved. While opting for a non-weighted maturity scoring approach offers simplicity, it may not fully reveal the nuanced distinctions between the sub-enablers. Future studies could play a crucial role in unraveling the potential benefits of adopting a weighted scoring approach.

### Limitations

Several limitations should be noted in our study. First, we did not include developers from different clinical pathways in the development of the matrix, which may limit the generalizability of our results. Additionally, we did not have a PFP in our CPCT, which could have provided valuable input from the patient perspective. To mitigate these limitations, we used an iterative consensus-based approach to gather input from a diverse group of stakeholders in developing the matrix.

We considered higher scores as improvements in pathways’ maturity. However, this may not be a reflection of reality. Currently, there is no gold standard by which to measure the accuracy of enablers or sub-enablers of our maturity matrix. At this stage, it was considered critical to ensure that stakeholders agreed on what enablers or sub-enablers were important to observe and how to differentiate between strong and weak performance in those attributes (i.e., content and face validity). Stakeholders agreed that all relevant elements of pathway maturity were included in the enablers and sub-enablers and that measurement trajectories were appropriate. Reviewing the CPP with the CPP development team leader seemed to confirm the face validity of the matrix because it was considered by a targeted end user as effective in measuring maturity (i.e., dynamic state of planning, development, and readiness for a pathway to be implemented, replicated or scaled up, and sustained in its intended clinical settings).

### Future direction

There are several areas for future research related to our clinical pathways maturity matrix. First, we did not perform test–retest or inter-rater reliability testing of the matrix, and therefore future studies should evaluate the matrix’s reliability and validity. Further evaluation is needed to determine if the matrix is able to predict pathway progression to future improved state and successful implementation. To do this, the CPCT will monitor pathway development and implementation using the matrix and whether additional elements of pathway maturity emerge with more widespread use of the matrix.

Second, our study highlights the need for standardized measures for performance management of pathways (e.g., length of stay, patient reported experience measures, and patient reported outcome measures). However, IT support is needed to access data. Future research should explore the data access barrier and examine its impact on pathway implementation.

Finally, future versions of the matrix could include implementation, service, and client outcomes, such as pathway adoption, sustainability, or stakeholder satisfaction [22]. These outcomes would provide a more comprehensive picture of the pathway's maturity and impact. It is worth noting that several tools can supplement the clinical pathways maturity matrix, adding complexity, sophistication, and efficacy to the evaluation process. While the focus of this study was on the development and testing of the matrix, it is important to acknowledge the value of these supplementary tools. Future research could explore the integration of these tools into the evaluation process and their impact on the accuracy and utility of the matrix.

## Conclusions

The SHA Clinical Pathways Core Team (CPCT) has developed a maturity matrix that can serve as a tool for evaluating both new and existing clinical pathways. This matrix plays a role in evaluating the design quality of pathways and identifying gaps and limitations in their implementation and replication. We believe that our matrix enables development and implementation teams to monitor clinical pathways over time to ensure they are achieving their intended effects at multiple levels, including the patient, provider, and system levels. This comprehensive evaluation warrants that clinical pathways align with their objectives and deliver value across the healthcare systems.

Further research will be necessary to determine the real-world impact of implementing this matrix. We aim to investigate whether utilizing the matrix leads to improved clinical pathways and whether it can effectively identify when a pathway is ready for implementation. By doing so, we hope to contribute to the ongoing improvement of clinical care, enhancing patient outcomes, provider satisfaction, and the efficiency of healthcare delivery.

### Supplementary Information

Supplementary Material 1.

Supplementary Material 2.

Supplementary Material 3.

Supplementary Material 4.

Supplementary Material 5.

Supplementary Material 6.

Supplementary Material 7.

Supplementary Material 8.

## Data Availability

No datasets were generated or analysed during the current study.

## Abbreviations

CPCT: Clinical Pathways Core Team
CPP: Chronic Pain Pathway
LHS: Learning Health Systems
MoH: Saskatchewan Ministry of Health
PCE: Patient and Client Experience
PFPs: Patient and Family Partners
QI: Quality Improvement
SHA: Saskatchewan Health Authority

## References

[1] Menear M, Blanchette MA, Demers-Payette O, Roy D (2019). A framework for value-creating learning health systems. Health Res Policy Syst.

[2] Committee on the Learning Health Care System in America, Institute of Medicine. Best Care at Lower Cost: The Path to Continuously Learning Health Care in America. Smith M, Saunders R, Stuckhardt L, McGinnis JM, editors. Washington (DC): National Academies Press (US); 2013. Available from: http://www.ncbi.nlm.nih.gov/books/NBK207225/. Cited 2023 May 3. 24901184

[3] Friedman C, Rubin J, Brown J, Buntin M, Corn M, Etheredge L (2015). Toward a science of learning systems: a research agenda for the high-functioning Learning Health System. J Am Med Inform Assoc.

[4] Allen C, Coleman K, Mettert K, Lewis C, Westbrook E, Lozano P (2021). A roadmap to operationalize and evaluate impact in a learning health system. Learn Health Syst.

[5] Foley T, Vale L (2023). A framework for understanding, designing, developing and evaluating learning health systems. Learn Health Syst.

[6] Gartner JB, Abasse KS, Bergeron F, Landa P, Lemaire C, Côté A (2022). Definition and conceptualization of the patient-centered care pathway, a proposed integrative framework for consensus: a Concept analysis and systematic review. BMC Health Serv Res.

[7] Seckler E, Regauer V, Rotter T, Bauer P, Müller M (2020). Barriers to and facilitators of the implementation of multi-disciplinary care pathways in primary care: a systematic review. BMC Fam Pract.

[8] Vanhaecht K, Panella M, Van Zelm R, Sermeus W (2010). An overview on the history and concept of care pathways as complex interventions. Int J Care Pathw.

[9] Kinsman L, Rotter T, James E, Snow P, Willis J (2010). What is a clinical pathway? Development of a definition to inform the debate. BMC Med.

[10] Lawal AK, Rotter T, Kinsman L, Machotta A, Ronellenfitsch U, Scott SD (2016). What is a clinical pathway? Refinement of an operational definition to identify clinical pathway studies for a Cochrane systematic review. BMC Med.

[11] Groot G, Ollegasagrem S, Khakpour M, Panahi A, Goodridge D, Lloyd J (2022). Facilitators and barriers to clinical pathway uptake and utilization among primary care providers in Saskatchewan - a qualitative study. Clin Invest Med.

[12] Flores EJ, Mull NK, Lavenberg JG, Mitchell MD, Leas BF, Williams A (2019). Using a 10-step framework to support the implementation of an evidence-based clinical pathways programme. BMJ Qual Saf.

[13] Schriek MB, Turetken O, Kaymak U. A maturity model for care pathways. In Proceedings of the European Conference on Information Systems (ECIS 2016), 12-15 June 2016, Istanbul, Turkey (pp. 1-16). Article Paper 127 Association for Information Systems. 2016.

[14] De Bleser L, Depreitere R, De Waele K, Vanhaecht K, Vlayen J, Sermeus W (2006). Defining pathways. J Nurs Manag.

[15] Coeckelberghs E, Verbeke H, Desomer A, Jonckheer P, Fourney D, Willems P (2021). International comparative study of low back pain care pathways and analysis of key interventions. Eur Spine J Off Publ Eur Spine Soc Eur Spinal Deform Soc Eur Sect Cerv Spine Res Soc.

[16] Agency for Healthcare Research and Quality. Six Domains of Health Care Quality. Available from: https://www.ahrq.gov/talkingquality/measures/six-domains.html. Cited 2023 May 3.

[17] Kolukısa Tarhan A, Garousi V, Turetken O, Söylemez M, Garossi S (2020). Maturity assessment and maturity models in health care: a multivocal literature review. Digit Health.

[18] Toy JM, Drechsler A, Waters RC (2018). Clinical pathways for primary care: current use, interest and perceived usability. J Am Med Inform Assoc JAMIA.

[19] Fischer F, Lange K, Klose K, Greiner W, Kraemer A (2016). Barriers and strategies in guideline implementation-a scoping review. Healthc Basel Switz.

[20] Moleman M, Jerak-Zuiderent S, van de Bovenkamp H, Bal R, Zuiderent-Jerak T (2022). Evidence-basing for quality improvement; bringing clinical practice guidelines closer to their promise of improving care practices. J Eval Clin Pract.

[21] Nilsen P (2015). Making sense of implementation theories, models and frameworks. Implement Sci.

[22] Proctor E, Silmere H, Raghavan R, Hovmand P, Aarons G, Bunger A (2011). Outcomes for implementation research: conceptual distinctions, measurement challenges, and research agenda. Adm Policy Ment Health.

[23] Home | SaskHealthAuthority. 2023. Available from: https://www.saskhealthauthority.ca/. Cited 2023 May 3.

[24] Clinical Pathways | SaskHealthAuthority. Available from: https://www.saskhealthauthority.ca/our-organization/quality-care-patient-safety/quality-improvement/stewardship-and-clinical-appropriateness/clinical-pathways. Cited 2023 May 3.

[25] Patient & Client Experience | SaskHealthAuthority. Available from: https://www.saskhealthauthority.ca/intranet/departments-programs/quality-safety-and-information/patient-client-experience. Cited 2023 May 16

[26] Patient & Family Centred Care | SaskHealthAuthority. Available from: https://www.saskhealthauthority.ca/our-organization/quality-care-patient-safety/patient-family-centred-care. Cited 2023 May 16.

[27] Saskatchewan Health Authority - Patient and Family Centered Care & SCPOR. Available from: https://app.betterimpact.com/PublicOrganization/e6802658-3612-4f9f-8be5-59963b262cbf/1. Cited 2023 May 16.

[28] Harris PA, Taylor R, Thielke R, Payne J, Gonzalez N, Conde JG (2009). Research electronic data capture (REDCap)–a metadata-driven methodology and workflow process for providing translational research informatics support. J Biomed Inform.

[29] Harris PA, Taylor R, Minor BL, Elliott V, Fernandez M, O’Neal L (2019). The REDCap consortium: Building an international community of software platform partners. J Biomed Inform.

[30] The leader in collaboration & customer experience | Webex. Webex by Cisco. Available from: https://www.webex.com/. Cited 2023 May 12.

[31] Mural is a collaborative intelligence company | Mural. Available from: https://www.mural.co/. Cited 2023 May 4.

