# Supplementary material for: Development and validity testing of a matrix to evaluate maturity of clinical pathways: a case study in Saskatchewan, Canada
Source: BMC Health Serv Res. 2024 Jul 10;24:793. doi: 10.1186/s12913-024-11239-x (PMC11234781; doi:10.1186/s12913-024-11239-x)
Supplement: Supplementary file 1 — Supplementary Material 1. [file 12913_2024_11239_MOESM1_ESM.docx]

**Supplementary File 1**

**Clinical Pathways Maturity Matrix Evaluation Survey**

Welcome to the Clinical Pathways Maturity Matrix Evaluation survey.

The purpose of the survey is to collect the insights of experts in the field of clinical pathways about the criteria required to evaluate the maturity of clinical pathways. We are seeking your feedback on the importance or relevance of a set of criteria (sub-enablers) to evaluate maturity of the SHA clinical pathways. Please rank each criterion (sub-enabler) from 1 (not important or relevant at all) to 10 (very important or relevant) when you consider evaluating maturity of a clinical pathway. Please complete the survey by July 15, 2022. If there are any questions or comments, please do not hesitate to contact clinical.pathway@saskhealthauthority.ca. We appreciate your time and support.

| Name (optional) |  |
| --- | --- |
| Email (optional) |  |
| Field of Practice (optional) |  |

| Criteria (sub-enablers): | | | | | | | | | | |
| --- | --- | --- | --- | --- | --- | --- | --- | --- | --- | --- |
|  | Not important or relevant at all (1) | (2) | (3) | (4) | (5) | (6) | (7) | (8) | (9) | Most important or relevant (10) |
| 1 Pathway Objective Alignment |  |  |  |  |  |  |  |  |  |  |
| 2 Pathway Definition |  |  |  |  |  |  |  |  |  |  |
| 3 Compliance |  |  |  |  |  |  |  |  |  |  |
| 4 Clarity in the Decision Criteria |  |  |  |  |  |  |  |  |  |  |
| 5 Design Approach |  |  |  |  |  |  |  |  |  |  |
| 6 Owner (Identity) |  |  |  |  |  |  |  |  |  |  |
| 7 Role Awareness/Role Functionality |  |  |  |  |  |  |  |  |  |  |
| 8 IT Infrastructure And Information Sharing |  |  |  |  |  |  |  |  |  |  |
| 9 Network of Pathways |  |  |  |  |  |  |  |  |  |  |
| 10 Metrics Alignment |  |  |  |  |  |  |  |  |  |  |
| 11 Metrics Use |  |  |  |  |  |  |  |  |  |  |
| 12 Structured Collection of Data |  |  |  |  |  |  |  |  |  |  |
| 13 Availability of Performance Information |  |  |  |  |  |  |  |  |  |  |
| 14 Availability/Accessibility Of Data |  |  |  |  |  |  |  |  |  |  |
| 15 Capacity Monitoring |  |  |  |  |  |  |  |  |  |  |
| 16 Stakeholder Engagement |  |  |  |  |  |  |  |  |  |  |
| 17 Clinician's Awareness And Contribution |  |  |  |  |  |  |  |  |  |  |
| 18 Adaptability |  |  |  |  |  |  |  |  |  |  |
| 19 External Maturity Evaluation |  |  |  |  |  |  |  |  |  |  |

| Comments / Feedback (optional): |  |
| --- | --- |
