# Supplementary material for: Development and validity testing of a matrix to evaluate maturity of clinical pathways: a case study in Saskatchewan, Canada
Source: BMC Health Serv Res. 2024 Jul 10;24:793. doi: 10.1186/s12913-024-11239-x (PMC11234781; doi:10.1186/s12913-024-11239-x)
Supplement: Supplementary file 2 — Supplementary Material 2. [file 12913_2024_11239_MOESM2_ESM.docx]

**Supplementary File 2**

**Maturity matrix enablers, sub-enablers, their definitions, and the three trajectory definitions**

**Date Completed:**

| Enabler | Sub-enabler | Definition | Explanation | Maturity Level | | | Notes, Comments, Rationale |
| --- | --- | --- | --- | --- | --- | --- | --- |
|  |  |  |  | **Low (0)** | **Moderate (1)** | **High (2)** |  |
| Design | Pathway Objective Alignment* | The degree by which the objective of the pathway is aligned to the objective of the care delivery to the specific patient group of the pathway. | Complete the "Pathway Alignment Tool" to understand the alignment between the objectives of the pathway and objectives of care delivery.  Ensure the objectives of the pathway and the objectives of care delivery meet the needs of patients. | The pathway alignment tool has not been completed and the degree to which pathway objectives are aligned to the objectives of care delivery cannot be assessed. | The pathway alignment tool has been completed but not all of the pathway objectives are in alignment with the objectives of care delivery. | The pathway alignment tool has been completed and all of the pathway objectives are in alignment with the objectives of care delivery. |  |
|  | Pathway Definition* | The degree in which the design of the pathway is defined with a clear structure, terminology, and roles. | Structural Components - Using the prototype checklist to identify the structural components of a typical pathway, which includes - Prevention, Assessment, and Clinical Management.  Terminology - Clarifies any language that may not be known to all users.  Roles - Clarity between all stakeholder roles within the pathway (know when to use the pathway and understand who does what within the pathway). There are defined roles of the health care team members in the patient's journey map or algorithm.  This sub-enabler is evaluating the pathway from the perspective of the "developers" of the pathway. | The structural components in the pathway (prevention, assessment, clinical management) have been identified but the terminology and roles among pathway stakeholders are not clearly defined. | The structural components in the pathway (prevention, assessment, clinical management) have been identified, and either pathway content clarifies all terminology or the roles among pathway stakeholders are clearly defined. | The structural components in the pathway (prevention, assessment, clinical management) have been identified, pathway content clarifies all terminology and roles among pathway stakeholders are clearly defined. |  |
|  | Compliance | The degree in which a pathway is designed, taking into consideration integrated policies, best clinical practice guidelines, evidence, and legislation. | The Clinical Pathway Development Record has been populated to identify that there is documentation of the evidence used during pathway development.  The pathway team has integrated policies, best clinical practice guidelines, evidence, legislation, Accreditation Canada standards and Choosing Wisely Canada recommendations in the clinical pathway development. | The Clinical Pathway Development Record has not been completed. | The Clinical Pathway Development Record has been partially completed, either documentation of a literature search including research articles, policies, best clinical practice guidelines, legislation, or other evidence has been documented or metrics have been documented. | The Clinical Pathway Development Record has been fully completed. |  |
|  | Clarity in the Decision Criteria | There is sufficient detail in the decision moments and in the decision criteria in the design of the pathway. | Decision Moments - There is clarity between sections as to when to refer and what the criteria is for both clinical referral and clinical management of the patient (e.g., when to send a patient for lab testing, imaging, follow ups).  When a clinician moves between the elements - from Prevention to Assessment to Clinical Management and within Clinical Management (from Diagnosis to Treatment to Follow Up), connections between these elements should be clear within the pathway design and the moments should be connected based on evidence.  Decision Criteria - Decisions are based on evidence or data, not personal decisions, and are clear. | Decision moments and decision criteria for referral and clinical management have not been outlined in the design of the pathway. | Some but not all decision moments and decision criteria for referral and clinical management have been outlined in the design of the pathway. | All decision moments and decision criteria for referral and clinical management have been outlined in the design of the pathway. |  |
|  | Patient Journey Map** | There is sufficient detail that has been included in the patient journey map or algorithm that outlines all of the patient touchpoints that occur within the pathway. | Patient Touchpoint - Touchpoints can include any interaction between the patient and provider, the health care system, community support services, community resources, or where a decision in patient care is made.  Patient Journey Map is a visual representation of the various stages and touchpoints that a patient may go through when seeking healthcare services. It outlines the typical steps a patient may take, from the initial search for healthcare services to the ultimate goal of achieving optimal health outcomes.  Elements of the patient journey map should include the patient's touchpoints from Prevention to Assessment and Clinical Management, incorporating the referral process. | A patient journey map or algorithm has not been developed. | Some but not all patient touch points have been outlined in the patient journey map or algorithm. | All patient touch points have been outlined in the patient journey map or algorithm. |  |
|  | Design Approach | The degree in which a structured approach (e.g., a reference framework) is used and different stakeholders were involved during the design of the pathway (from primary care to specialized / hospital based care). | Structured Approach - The pathway development team used the Pathway Framework and the Pathway Prototype Checklist to design the pathway.  Different Stakeholders - As outlined within the clinical pathway definition, it is required to involve a multidisciplinary group of stakeholders, including patient partners, during the design of the pathway. | Neither the Pathway Framework nor the Pathway Prototype Checklist was used during the design of the pathway. A group of stakeholders were involved in the design of the pathway but involvement was at a local level. | Both the Pathway Framework and the Pathway Prototype Checklist were used during the design of the pathway. A diverse group of provincial stakeholders, not including patient family partners were involved in the design of the pathway. | Both the Pathway Framework and the Pathway Prototype Checklist were used during the design of the pathway. A diverse group of provincial stakeholders, including patient family partners were involved in the design of the pathway. |  |
| Owner and Performer | Owner (Identity)* | The extent to which the pathway ownership structure is effective in improving the pathway performance. | Ownership Structure - The sponsor, operational owner(s), and clinical owners are identified, roles are clearly defined and they are actively participating in the ongoing development of and monitoring of the pathway. These stakeholders hold a position that can affect and support change.  Pathway Performance is impacted by multiple factors including implementation, replication, ongoing monitoring and updates (e.g., reviewing guidelines), and sustainability (long- and short-term measurement). | The sponsor, operational owners, and clinical owners have not been identified. | The sponsor, operational owners, and clinical owners have been identified. These stakeholders are not actively participating in the ongoing development and monitoring of the pathway. | The sponsor, operational owners, and clinical owners have been identified. These stakeholders are actively participating in the ongoing development and monitoring of the pathway. |  |
|  | Role Awareness/Role Functionality | The degree in which a pathway participant has awareness of his/her part in the pathway and the ability to perform his/her task as described in the pathway design. | Role Awareness - Clinicians understand their role and know how their clinical decisions (decision moments) impact the whole patient journey.  Mechanisms - These are types of data collection tools (e.g., surveys, questionnaires, interviews, focus groups) used as proxies to assess role awareness and role functionality of pathway participants.  This sub-enabler is evaluating the pathway from the perspective of the "end users" of the pathway. | There are no measures or mechanisms in place to assess:  (1) Pathway participant role awareness or  (2) Ability to perform their tasks in the pathway. | There are measures and mechanisms in place to assess either:  (1) Pathway participant role awareness or  (2) Ability to perform their tasks in the pathway. | There are measures and mechanisms in place to assess:  (1) Pathway participant role awareness and  (2) Ability to perform their tasks in the pathway. |  |
| Infrastructure | IT Infrastructure and Information Sharing | The degree by which IT infrastructure facilitates the sharing of materials and information across both internal and external data systems | Facilitates - The extent to which IT infrastructures (internal and external) are either connected or not connected, in order to facilitate the sharing of materials and information  Materials/Information - Some examples, among many, include referral forms, order sets, work standards, website links, prescription pads and pathway metrics  Internal Data Systems - SHA information systems and databases  External Data Systems - Non SHA information systems and data bases (MoH data, private clinic EMRs, community data systems - pharmacies home care or long term care facilities)  Information sharing across data systems - Refers to the process of exchanging data or information between two or more separate systems, applications, or databases and to enable different systems to work together seamlessly. | Internal and external IT infrastructures are not connected and information sharing is not occurring across either internal or external data systems. | Internal and external IT infrastructures are not connected but information sharing is occurring across either internal or external data systems. | Internal and external IT infrastructures are connected and information sharing is occurring across both internal and external data systems. |  |
|  | Network of Pathways | The degree to which a pathway is interconnected to other pathways that have overlapping clinical problems. | Refer to the completed "Clinical Pathway Listing" template. This document should be populated during the development of a clinical pathway to understand the alignment and interconnectedness between existing pathways and the new pathway currently being developed. | Interconnected pathways have not been identified in the "Clinical Pathway Listing" template. | Interconnected pathways have been identified in the "Clinical Pathway Listing" template but the "briefly describe" column is incomplete for all identified pathways. | Interconnected pathways have been identified in the "Clinical Pathway Listing" template and the "briefly describe" column is complete for all identified pathways. |  |
| Performance Management | Metrics Alignment | The degree in which pathway metrics (i.e., process, outcome, and balancing measures) are uniformly defined, and pathway objectives (e.g., patient and provider experience) have been considered in the development of the metrics. | Uniformly defined - There are two sets of measures, (1) measures specific to a single pathway (2) measures that can be applied to all pathways.  NOTE: Pathway objectives should be considered in the development of pathway metrics.  Regardless of the measures selected, it is important the metric(s) are defined and worded in the same way so measurement is consistent. | The pathway does not have uniformly defined measures. | The pathway has uniformly defined measures but not all measures are aligned with the pathway objectives. | The pathway has uniformly defined measures and all measures align with the pathway objectives. |  |
|  | Structured Collection of Data | The degree in which a structured data collection plan is in place (including what is measured, in which setting, how will it be measured, by whom and by when - frequency/timeframe). | Structured Data Collection Plan - The plan would include:  - Selected measures  - How the measures are calculated (or defined)  - How data will be collected (method- tools/templates used)  - How often data will be collected  - Who would collect the data | A structured data collection plan is not in place. | A structured data collection plan is in development. | A structured data collection plan is in place. |  |
|  | Availability/Accessibility of Data | The degree in which the availability and accessibility of pathway data facilitates the development of metrics. | Availability - Processes in place to collect and store data in standardized methods.  Accessibility - Processes in place to facilitate retrieval, modification and manipulation of on demand data from IT systems to the users of the data. | Data are unavailable and/or inaccessible. | Data are available and accessible but are not being used in pathway metrics development. | Data are available and accessible and are being used in pathway metrics development. |  |
|  | Metrics Use | The degree in which the pathway metrics (i.e., process, outcome, and balancing measures) are effectively used to improve the achieved performance. | The process, outcome, and balancing measures are used in Plan, Do, Check, Act (PDCA) cycles to identify quality improvement opportunities and improve pathway performance. | The pathway metrics (process, outcome, and balancing measures) have not been developed. | The pathway metrics (process, outcome, and balancing measures) have been developed but they are not being used to improve pathway performance. | The pathway metrics (process, outcome, and balancing measures) have been developed and they are being used to improve pathway performance. |  |
|  | Availability of Performance Information | The degree in which pathway metrics (i.e., process, outcome, and balancing measures) are available, shared and translated into something that stakeholders can understand. | Targets how the performance information is being shared and communicated to all stakeholders. Translating the generated data from the pathway into infographics that all stakeholders can understand. | The pathway metrics (process, outcome, and balancing measures) are not available to stakeholders. | The pathway metrics (process, outcome, and balancing measures) are available to stakeholders but not shared and translated in a format that is easy for them to understand. | The pathway metrics (process, outcome, and balancing measures) are available to stakeholders and are shared and translated in a format that is easy for them to understand. |  |
|  | Capacity Monitoring | The degree by which there is adequate allocations of key resources, such as facilities, equipment, and human resources, and these allocations are monitored. | Measuring Allocation - What does the pathway need provincially vs. what the pathway did or did not get (facilities, equipment, and human resources). It is important to understand allocations across the province and how these allocations fit into each of the provincial health networks.  Monitoring - How often are the allocations, required to implement the pathway, as intended, being monitored?  Are they continually available?  NOTE: The "Capacity Monitoring Mural Board" will be used to monitor adequate allocation of facilities, equipment and human resources. It is important that the Mural board is completed across the provincial health networks so that all areas of the province are considered when developing a pathway. Baseline allocations that have been populated on the "Capacity Monitoring Mural Board" should be used as a reference to see if the baseline has changed over time. If the baseline has changed, a newer version of the Mural board will be considered as a reference. | The "Capacity Monitoring Mural board" has not been completed. | The "Capacity Monitoring Mural board" has been completed but the allocations are not being monitored. | The "Capacity Monitoring Mural board" has been completed and the allocations are being monitored. |  |
| Culture | Pathway Awareness | The degree in which mechanisms are in place to raise stakeholders' (e.g., patients, clinicians, etc.) awareness of the pathway. | Mechanisms - May include emails, surveys, social media, SHA News, presentations, wall walks, department meetings, Saskatchewan Medical Association (SMA) or other professional newsletters, or other methods. | There are no mechanisms developed to raise awareness of the pathway. | There are mechanisms developed to raise awareness of the pathway but the mechanisms have either not been implemented or have been partially implemented. | There are mechanisms developed to raise awareness of the pathway and the mechanisms have been fully implemented. |  |
|  | Ongoing Stakeholder Engagement | The degree in which stakeholders, including patient partners, are engaged to provide ongoing contributions for change to improve the pathway structure and its processes. | Refer to the IAP2/PORLET 2.0/SHA Engagement framework to understand the level of engagement; the difference between inform, consult, involve, collaborate and empower.  Inform - Stakeholders and patient family partners do not have any direct role in any decision making.  Involve - Stakeholders and patient family partners consistently share ideas that are sometimes reflected in project decisions.  Collaborate- Stakeholders and patient family partners co-develop ideas that are incorporated into project decisions.  Ongoing Contributions - there is continuous engagement of stakeholders and patient and family partners, using multiple strategies (e.g., audit and feedback) throughout various stages of pathway maturity to improve the pathway structure and its processes. The strategies would depend on the level of stakeholder engagement and the maturity of the pathway. | There is either no ongoing engagement of stakeholders and patient and family partners or they are informed to provide ongoing contributions for change to improve the pathway structure and its processes. | Stakeholders and patient and family partners are involved to provide ongoing contributions for change to improve the pathway structure and its processes. | Stakeholders and patient and family partners collaborate to provide ongoing contributions for change to improve the pathway structure and its processes. |  |
|  | Adaptability | The degree in which the pathway is designed with the adaptability for implementation and replication across multiple settings and changes are tracked within each setting when this occurs. | "Adaptability" is acceptable (for both clinicians and processes) within the implementation / replication of the pathway in various local settings. While adaptability is acceptable, pathway stakeholders must ensure these changes will not impact the integrity of the pathway design and level of patient care intended.  Changes are tracked - By utilizing the replication checklist to ensure:  (1) Documentation is available for sharing with other users for replication  (2) This documentation is retained for all settings in which replication occurs. | The replication checklist has not been completed, the pathway has been implemented in one local setting but has not been replicated provincially. | The replication checklist has been completed, the pathway has been implemented in more than one local setting but has not been replicated provincially. | The replication checklist has been completed, the pathway has been fully implemented / replicated provincially. |  |
|  | External Maturity Evaluation | The degree in which the pathway is audited for maturity by an independent SHA governing body. | An independent team, such as the Pathway Oversight Committee, would review the maturity of each pathway annually (audit), to see if it is progressing from the pathway's baseline score, using the maturity matrix scoring system.  Independent SHA governing body - An "external team" that was not part of the development, piloting and implementation phases of the pathway (e.g., Pathway Oversight Committee). | The pathway is not being audited by an independent SHA governing body. | The pathway is being audited by an independent SHA governing body, but audits do not occur annually. | The pathway is being audited by an independent SHA governing body and audits occur annually. |  |
| * Original definitions are adapted from Schriek et al. (1)  ** Patient Journey Map was added as a new sub-enabler to the maturity matrix. | | | | | | |  |

1. Schriek M, Turetken O, Kaymak U. A Maturity model for care pathways. In: The European Conference on Information Systems (ECIS2016). Istanbul,Turkey; 2016. p. 1–16.
