# Supplementary material for: Development and validity testing of a matrix to evaluate maturity of clinical pathways: a case study in Saskatchewan, Canada
Source: BMC Health Serv Res. 2024 Jul 10;24:793. doi: 10.1186/s12913-024-11239-x (PMC11234781; doi:10.1186/s12913-024-11239-x)
Supplement: Supplementary file 3 — Supplementary Material 3. [file 12913_2024_11239_MOESM3_ESM.docx]

**Supplementary File 3**

**Clinical Pathway Alignment Tool**

**Date Completed:**

**PURPOSE OF THIS DOCUMENT:** When developing a new clinical pathway, this form must be completed by pathway developers. Completion of this template will determine the degree to which the objectives of the clinical pathway align with the objectives of care delivery.

**NOTE:** The information populated in this template will inform the measurement on the "Maturity Matrix" for the sub-enabler "Pathway Objective Alignment." This measurement will determine whether there is alignment between the objectives of the pathway and objectives of care delivery.

Instruction:

1. List objectives of care delivery in the first table
2. List objectives of the pathway in the second table
3. Match each pathway objective to one or more care delivery objectives. Enter the “objective number” from the first table into the “matching objective number” of the second table

| **Objective Number** | **OBJECTIVES OF CARE DELIVERY** |
| --- | --- |
|  |  |
|  |  |
|  |  |
|  |  |
|  |  |

| **Matching Objective Number** | **OBJECTIVES OF THE PATHWAY** |
| --- | --- |
|  |  |
|  |  |
|  |  |
|  |  |
|  |  |
