# Supplementary material for: Development and validity testing of a matrix to evaluate maturity of clinical pathways: a case study in Saskatchewan, Canada
Source: BMC Health Serv Res. 2024 Jul 10;24:793. doi: 10.1186/s12913-024-11239-x (PMC11234781; doi:10.1186/s12913-024-11239-x)
Supplement: Supplementary file 4 — Supplementary Material 4. [file 12913_2024_11239_MOESM4_ESM.docx]

**Supplementary File 4**

**Clinical Pathway Development Record**

**Date Completed:**

Purpose of this tool is to track the changes and decisions made during the development of a clinical pathway. The key information is what the situation was before the change, what the change was, how the change improved things for users of the pathway (i.e., patients, families, staff, etc.), and who were involved in the change. Sharing the changes, ideas, and thought processes using the clinical pathway development record enhances spreading learning and knowledge transfer across settings, improves communication among teams, and motivates further replication or scaling of the clinical pathway implementation.

| **Pathway Owner Name:** | | |  | | | |
| --- | --- | --- | --- | --- | --- | --- |
| **Job Title:** | | |  | | | |
| **Location (Dept./Area):** | | |  | | | |
| **Contact Number:** | | |  | | | |
| **Pathway Publication Date:**  (Dated version of the pathway that will be implemented) | | |  | | | |
|  | | | | | | |
| **Identify Key Stakeholders:**  Pathway development includes keeping a record of all key stakeholders. Use the template “Clinical Pathway Stakeholder Feedback Log” to record this information. Check the box below to indicate this has been completed.  Have identified key stakeholders using “Clinical Pathway Stakeholder Feedback Log” template | | | | | | |
| **References**  **References in this section may include: research articles, policies, best clinical practice guidelines, legislation or other evidence** | | | | | | |
|  | **Title Of Publication** | **Author(s)** | | **Place Of Publication (Publisher)** | **Publication Date** | **Website Address (If Applicable)** |
| 1 |  |  | |  |  |  |
| 2 |  |  | |  |  |  |
| 3 |  |  | |  |  |  |
| 4 |  |  | |  |  |  |
| 5 |  |  | |  |  |  |
| **Measurement – Key Metrics**  **PILOT (90 Days Or Greater)** | | | | | | |
| 1 |  | | | | | |
| 2 |  | | | | | |
| 3 |  | | | | | |
| 4 |  | | | | | |
| 5 |  | | | | | |
| **Ongoing Measurement – Key Metrics** | | | | | | |
| 1 |  | | | | | |
| 2 |  | | | | | |
| 3 |  | | | | | |
| 4 |  | | | | | |
| 5 |  | | | | | |
