# Supplementary material for: Development and validity testing of a matrix to evaluate maturity of clinical pathways: a case study in Saskatchewan, Canada
Source: BMC Health Serv Res. 2024 Jul 10;24:793. doi: 10.1186/s12913-024-11239-x (PMC11234781; doi:10.1186/s12913-024-11239-x)
Supplement: Supplementary file 5 — Supplementary Material 5. [file 12913_2024_11239_MOESM5_ESM.docx]

**Supplementary File 5**

**Clinical Pathway Prototype Checklist**

**Date Completed:**

| Prototype Elements | Considered (Yes \| No) |
| --- | --- |
| PREVENTION | |
| Screening |  |
| ASSESSMENT | |
| History Taking And Physical Examination |  |
| Risk Factors |  |
| Testing And Evaluation |  |
| Assessment Forms |  |
| Diagnosis |  |
| CLINICAL MANAGEMENT | |
| Treatment |  |
| Order Sets And Checklists |  |
| Communication Plan (For Providers To Deliver Results To Patients) |  |
| Referrals |  |
| Virtual Care Points |  |
| Follow Up (Consider Evidence Based Timelines) |  |
| Clinical/Best Practice Guidelines |  |
| CLINICAL MANAGEMENT (PROVIDER RESOURCES) | |
| Provider Education |  |
| CLINICAL MANAGEMENT (PATIENT RESOURCES) | |
| Patient/Family Resources - (Patient Friendly Terminology) |  |
| Patient/Family Education |  |
| Pamphlets And Public Handouts |  |
| Clinics And Related Pathway Connections |  |
| Support Groups And Counselling |  |
| Multi-Cultural Considerations |  |
| VISUALS | |
| Patient Flow Map |  |
| Algorithms |  |
