# Supplementary material for: Development and validity testing of a matrix to evaluate maturity of clinical pathways: a case study in Saskatchewan, Canada
Source: BMC Health Serv Res. 2024 Jul 10;24:793. doi: 10.1186/s12913-024-11239-x (PMC11234781; doi:10.1186/s12913-024-11239-x)
Supplement: Supplementary file 6 — Supplementary Material 6. [file 12913_2024_11239_MOESM6_ESM.docx]

**Supplementary File 6**

**Clinical Pathway Listing**

**Date Completed:**

**PURPOSE OF THIS DOCUMENT:** When developing a new clinical pathway, this form must be completed by pathway developers to review and consult the list below. The Clinical Pathway Listing is a reference list of all the pathways in the province, including those currently in development. Completion of this template will identify areas where the new clinical pathway interconnects with existing pathways.

**NOTE:** The information populated in this template will inform the measurement on the "Maturity Matrix" for the sub-enabler "Network of Pathways." This measurement will determine whether interconnections to other pathways have been taken into consideration during development.

| **Is This Connected To The Current Pathway Being Developed** | **Existing Pathway** | **If Yes, Briefly Describe How The Current Pathway Being Developed Is Interconnected** |
| --- | --- | --- |
| Yes/No | Acute Stroke Pathway |  |
| Yes/No | Bariatric Surgery Pathway |  |
| Yes/No | Cancer Treatment Pathway |  |
| Yes/No | COPD Pathway |  |
| Yes/No | Chronic Pain Pathway |  |
| Yes/No | Diabetes Pathway |  |
| Yes/No | Fertility Care Pathway |  |
| Yes/No | Hip And Knee Pathway |  |
| Yes/No | Long COVID |  |
| Yes/No | Lower Extremity Wound Pathway |  |
| Yes/No | Multiple Sclerosis Pathway |  |
| Yes/No | Pelvic Floor Pathway |  |
| Yes/No | Prostate Cancer Pathway |  |
| Yes/No | Spine Pathway |  |
