# Supplementary material for: Development and validity testing of a matrix to evaluate maturity of clinical pathways: a case study in Saskatchewan, Canada
Source: BMC Health Serv Res. 2024 Jul 10;24:793. doi: 10.1186/s12913-024-11239-x (PMC11234781; doi:10.1186/s12913-024-11239-x)
Supplement: Supplementary file 7 — Supplementary Material 7. [file 12913_2024_11239_MOESM7_ESM.docx]

**Supplementary File 7**

**Capacity Monitoring**

**Date Completed:**

| Service Area #1 | | | |
| --- | --- | --- | --- |
| Would Be Needed To Implement The Clinical Pathway (List Things Beyond The Existing Budgetary Allocation) | | | |
| ADDITIONAL HUMAN RESOURCES | e.g., Specialty Services - Specialists, Physicians, Multi-disciplinary Teams/Clinicians, Pharmacists, Patient Family Partners, etc. |  |  |
| NEW EQUIPMENT | e.g., Lab And Imaging Equipment |  |  |
| HIGH COST MEDICATIONS OR PHARMACEUTICAL SERVICES |  |  |  |
| BUSINESS INFRASTRUCTURE | e.g., Required Facilities, Buildings, Specialized Clinics, Office Space, Public Facilities (Rental Of Space Or Development Of New Space) |  |  |
| IT INFRASTRUCTURE |  |  |  |
| TRAINING & EDUCATION |  |  |  |
| OTHER FACTORS |  |  |  |

| Service Area #2 | | | |
| --- | --- | --- | --- |
| Would Be Needed To Implement The Clinical Pathway (List Things Beyond The Existing Budgetary Allocation) | | | |
| ADDITIONAL HUMAN RESOURCES | e.g., Specialty Services - Specialists, Physicians, Multi-disciplinary Teams/Clinicians, Pharmacists, Patient Family Partners, etc. |  |  |
| NEW EQUIPMENT | e.g., Lab And Imaging Equipment |  |  |
| HIGH COST MEDICATIONS OR PHARMACEUTICAL SERVICES |  |  |  |
| BUSINESS INFRASTRUCTURE | e.g., Required Facilities, Buildings, Specialized Clinics, Office Space, Public Facilities (Rental Of Space Or Development Of New Space) |  |  |
| IT INFRASTRUCTURE |  |  |  |
| TRAINING & EDUCATION |  |  |  |
| OTHER FACTORS |  |  |  |

| Service Area #3 | | | |
| --- | --- | --- | --- |
| Would Be Needed To Implement The Clinical Pathway (List Things Beyond The Existing Budgetary Allocation) | | | |
| ADDITIONAL HUMAN RESOURCES | e.g., Specialty Services - Specialists, Physicians, Multi-disciplinary Teams/Clinicians, Pharmacists, Patient Family Partners, etc. |  |  |
| NEW EQUIPMENT | e.g., Lab And Imaging Equipment |  |  |
| HIGH COST MEDICATIONS OR PHARMACEUTICAL SERVICES |  |  |  |
| BUSINESS INFRASTRUCTURE | e.g., Required Facilities, Buildings, Specialized Clinics, Office Space, Public Facilities (Rental Of Space Or Development Of New Space) |  |  |
| IT INFRASTRUCTURE |  |  |  |
| TRAINING & EDUCATION |  |  |  |
| OTHER FACTORS |  |  |  |

| Service Area #4 | | | |
| --- | --- | --- | --- |
| Would Be Needed To Implement The Clinical Pathway (List Things Beyond The Existing Budgetary Allocation) | | | |
| ADDITIONAL HUMAN RESOURCES | e.g., Specialty Services - Specialists, Physicians, Multi-disciplinary Teams/Clinicians, Pharmacists, Patient Family Partners, etc. |  |  |
| NEW EQUIPMENT | e.g., Lab And Imaging Equipment |  |  |
| HIGH COST MEDICATIONS OR PHARMACEUTICAL SERVICES |  |  |  |
| BUSINESS INFRASTRUCTURE | e.g., Required Facilities, Buildings, Specialized Clinics, Office Space, Public Facilities (Rental Of Space Or Development Of New Space) |  |  |
| IT INFRASTRUCTURE |  |  |  |
| TRAINING & EDUCATION |  |  |  |
| OTHER FACTORS |  |  |  |
