# Supplementary material for: Development and validity testing of a matrix to evaluate maturity of clinical pathways: a case study in Saskatchewan, Canada
Source: BMC Health Serv Res. 2024 Jul 10;24:793. doi: 10.1186/s12913-024-11239-x (PMC11234781; doi:10.1186/s12913-024-11239-x)
Supplement: Supplementary file 8 — Supplementary Material 8. [file 12913_2024_11239_MOESM8_ESM.docx]

**Supplementary File 8**

**Replication Documentation Checklist**

**Date Completed:**

**Note:** Replication package can be available for other sites or areas after initial pilot has been completed (90 days or greater).

| Item | Stored on Shared Drive or SharePoint Site |
| --- | --- |
| Approval Form |  |
| Stakeholder Contact List |  |
| Map Or Algorithm (Include Both Patient And Provider) |  |
| Work Standards/Standard Work |  |
| Order Sets / Referral Forms, Clinical Standards / Procedures, Assessments (Newly Developed / Revised) |  |
| Pamphlets and Public Handouts |  |
| Provider Educational Material |  |
| Patient Education Material |  |
| Patient Family Partner (PFP) Orientation Package |  |
| Lessons Learned (Compilation From First Area of Implementation To Last Area of Implementation) LIVE DOCUMENT |  |
| Metrics/Results - During Pathway Pilot |  |
| Metrics/Results - Sustainability (Short Term, Long Term) |  |
| Clinical Pathway Alignment Tool |  |
| Clinical Pathway Development Record |  |
| Clinical Pathway Prototype Checklist |  |
| Clinical Pathway Listing |  |
| Capacity Monitoring |  |
